# 1169. Unconjugated Multi-epitope Peptides Adjuvanted with ALFQ Induce Durable and Broadly Reactive Antibodies to Human and Avian Influenza Viruses

**DOI:** 10.1093/ofid/ofad500.1009

**Published:** 2023-11-27

**Authors:** Nimisha Rikhi, Clara J Sei, Mangala Rao, Richard F Schuman, Kellie Kroscher, Gary R Matyas, Kevin Muema, Camille Lange, Aba Assiaw-Dufu, Elizabeth Hussin, Ousman Jobe, Carl R Alving, Gerald W Fischer

**Affiliations:** Longhorn Vaccines & Diagnostics, LLC, Gaithersburg, Maryland; Longhorn Vaccines & Diagnostics, LLC, Gaithersburg, Maryland; Military HIV Research Program, Silver Spring, Maryland; Antibody and Immunoassay Consultants, Rockville, Maryland; Longhorn Vaccines & Diagnostics, LLC, Gaithersburg, Maryland; Walter Reed Army Institute of Research, Silver Spring, Maryland; Longhorn Vaccines & Diagnostics, LLC, Gaithersburg, Maryland; Henry M Jackson Foundation for the Advancement of Military Medicine, Bethesda, Maryland; Longhorn Vaccines & Diagnostics, LLC, Gaithersburg, Maryland; Henry M Jackson Foundation for the Advancement of Military Medicine, Bethesda, Maryland; Henry M Jackson Foundation for the Advancement of Military Medicine, Bethesda, Maryland; Walter Reed Army Institute of Research, Silver Spring, Maryland; Longhorn Vaccines and Diagnostics, LLC, Gaithersburg, Maryland

## Abstract

**Background:**

Traditional influenza vaccine production often leads to mismatch due to antigenic drift, thereby reducing protection. Alternate vaccine strategies that are peptide-based, nanoparticle-based or nucleic acid-based are being developed. Composite multi-epitope peptides provide a cost-effective and easily scalable strategy towards a universal influenza vaccine. In this study, we demonstrate that unconjugated, multi-epitope peptides formulated with the highly potent adjuvant Army Liposome Formulation with QS-21 (ALFQ) generate broadly reactive durable antibodies to human and avian influenza viruses when administered intramuscularly or intradermally.

**Methods:**

ICR mice were immunized with an unconjugated composite influenza peptide vaccine comprising HA+NA (Flu Pep11) and Matrix (M1/M2/M2e)+T-cell epitope (Flu Pep5906) formulated with ALFQ. A low-dose (1 µg) and high-dose (20 µg) of vaccine was administered intramuscularly or intradermally. Isotype-specific IgG titers to composite peptides, individual epitopes, and multiple strains of influenza A (H1N1, H3N2, H5N1), and B (Yamagata, Victoria) were analyzed by ELISA. Neutralizing titers against Group 1 and Group 2 viruses were determined using microneutralization assay.

**Results:**

A robust IgG1 and IgG2b antibody response was seen at day 21 post primary immunization and remained steady for all groups up to day 200 (duration of study). IgG2a and IgG3 titers were also generated. Antibodies recognized multiple strains each of human and avian influenza A and influenza B virus subtypes, 200 days post primary immunization, and were comparable between doses and routes. Strong neutralizing titers were shown against Group 1 and 2 influenza viruses.

**Conclusion:**

An unconjugated peptide vaccine comprising multiple highly conserved epitopes of HA, NA and Matrix formulated with ALFQ adjuvant and administered intramuscularly or intradermally, generated broad and durable antibodies to human and avian influenza viruses. Induced antibodies neutralized seasonal and pandemic influenza strains. These composite peptides adjuvanted with ALFQ generate durable and broad immunity and provide a cost-effective and easily scalable strategy that moves us closer to a universal influenza vaccine.

**Disclosures:**

**All Authors**: No reported disclosures

